# Modulation of NMDA Receptor and TRPM4 Activity in Hippocampal Neurons with the NMDA Receptor/TRPM4 Interface Inhibitor Brophenexin

**DOI:** 10.1007/s12640-026-00788-0

**Published:** 2026-03-06

**Authors:** Jordan Casby, Rachel K. Allen, Ezequiel Marron Fernandez de Velasco, Stanley A. Thayer

**Affiliations:** Department of Pharmacology, University of Minnesota Medical School, Minneapolis, MN USA

**Keywords:** Excitotoxicity, NMDA receptor, TRPM4, Neuroprotection, Ca^2+^ signaling

## Abstract

Excitotoxic signaling mediated by *N*-methyl-D-aspartate receptors (NMDARs) is inhibited by NMDAR/TRPM4 complex inhibitors such as brophenexin (BPN). We used rat hippocampal neurons grown in culture to determine the effects of BPN on NMDAR and TRPM4 function. NMDA evoked concentration-dependent increases in intracellular Ca^2+^ that were inhibited by 10 µM BPN in a non-competitive manner. In contrast, the TRPM4 inhibitor 4-chloro-2-(2-(naphthalene-1-yloxy) acetamido) benzoic acid (NBA) increased the potency of NMDA at 22 °C. BPN inhibition of NMDAR-mediated increases in Ca^2+^ was fully reversible and recovered by rapid (30 s) and slow (90 min) processes. The rapid phase of recovery from BPN inhibition was mediated by trafficking through recycling endosomes as indicated by blockade of this phase with bafilomycin A1 an agent that prevents endosomal acidification. The full recovery of NMDAR function observed 90 min after washout of BPN was not affected by this treatment. Immunocytochemistry experiments suggested that BPN did not directly alter NMDAR trafficking but instead changes in surface and internal GluN2B immunoreactivity were likely homeostatic responses to inhibition of NMDAR function by BPN. In the presence of MK-801 to block NMDARs, 10 µM NBA inhibited spontaneous network-driven Ca^2+^ spiking by 74 ± 11% whereas 10 µM BPN reduced activity by 49 ± 6%. Thus, BPN inhibits TRPM4-dependent activity. In summary, BPN is a non-competitive, fully reversible inhibitor of NMDAR-mediated Ca^2+^ influx and produces a modest inhibition of TRPM4 function. Functional inhibition of these ion channels likely contributes to the neuroprotective properties of NMDAR/TRPM4 interface inhibitors.

**Graphical Abstract:**

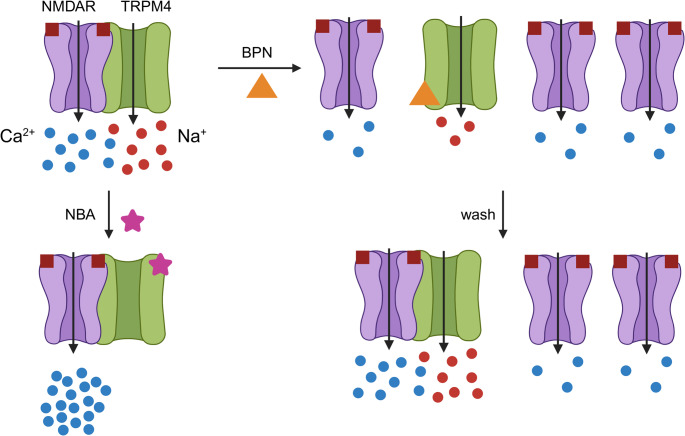

**Supplementary Information:**

The online version contains supplementary material available at 10.1007/s12640-026-00788-0.

## Introduction

Ca^2+^ influx through *N*-methyl-D-aspartate receptors (NMDARs) triggers signaling cascades important for learning and memory, network development, and gene expression (Hardingham et al. 2002; Ma et al. 2023a). Under excitotoxic conditions Ca^2+^ entry via NMDARs activates cell death pathways that underlie neurodegenerative disorders and traumatic brain injuries (Hardingham and Bading 2002). Several strategies to develop neuroprotective drugs by targeting pathological while sparing physiological NMDAR signaling have been pursued. NMDARs are tetramers composed of 2 GluN1 and 2 GlunN2 subunits (Vyklicky et al. 2014). GluN2 subunits vary in structure and have distinct properties (Haddow et al. 2022). NMDARs found at the synapse (sNMDARs) are preferentially GluN2A-containing and responsible for physiological signaling while activation of extrasynaptic NMDARs (esNMDARs), preferentially GluN2B-containing, trigger excitotoxic cell death. To date, no compounds highly selective for GluN2B-containing NMDARs have proved useful for treating neurodegenerative disorders (Lipton 2006; Ramírez et al. 2025). Targeting NMDARs based on subcellular location requires that NMDARs in the two environments possess different structural or functional properties. Recent identification of NMDAR binding partners has presented a new target for selective attenuation of pathological NMDAR signaling.

NMDAR binding to other ion channel subunits dramatically affects NMDAR function. The α2δ−1 subunit of the voltage-gated Ca^2+^ channel binds to and enhances NMDAR-mediated Ca^2+^ influx and is the target of gabapentinoid drugs used for treating neuropathic pain (Huang et al. 2025). α2δ−1 participates in NMDAR trafficking and plays a key role in membrane insertion (Zhou et al. 2021). Transient receptor potential melastatin 2 (TRPM2) is a Ca^2+^-permeable ion channel that forms a complex with the NMDAR and is implicated in excitotoxic cell death. Under ischemic conditions TRPM2 increases NMDAR phosphorylation by protein kinase C gamma (PKCγ) by bringing the kinase in close apposition to the NMDAR thus enhancing its Ca^2+^-dependent activation (Zong et al. 2025). Phosphorylation by PKCγ increases NMDAR activity and surface expression of the NMDAR/TRPM2 complex during ischemia. NMDAR/TRPM2 interface inhibition is neuroprotective (Zong et al. 2025). TRPM4 is a ubiquitously expressed monovalent cation channel activated by increases in intracellular free Ca^2+^ concentration ([Ca^2+^]_i_). An important physiological role of TRPM4 in neurons is to enhance dendritic excitability by producing a depolarizing Na^+^ flux (Menigoz et al. 2016; Li et al. 2021). TRPM4 preferentially binds to esNMDARs to modulate NMDAR iontropic activity andNMDAR-mediated increases in [Ca^2+^]_i_ (Yan et al. 2020; Casby et al. 2024). Disrupting the NMDAR/TRPM4 interface is neuroprotective, presumably by disrupting the Ca^2+^-dependent mitochondrial depolarization that results from activation of esNMDARs (Yan et al. 2020).

A new class of drugs bind to the NMDAR/TRPM4 interface to disrupt the complex (Yan et al. 2020) and inhibit Ca^2+^ influx through the NMDAR (Casby et al. 2024). Compounds such as brophenexin (BPN)(Yan et al. 2020), FP802 (Yan et al. 2024), and HZS60 (Sun et al. 2025) provide neuroprotection both in cell culture and in rodent models of stroke and amyotrophic lateral sclerosis (ALS). These drugs appear to be well tolerated, presumably because they are primarily interfering with esNMDAR-mediated Ca^2+^ influx and thus, spare physiological Ca^2+^ signaling mediated by sNMDARs (Yan et al. 2020).

Here, we examine the effects of the NMDAR/TRPM4 interface inhibitor BPN on NMDA-evoked [Ca^2+^]_i_ responses in hippocampal neurons in culture. We show that BPN and the TRPM4 inhibitor 4-chloro-2-(2-(naphthalene-1-yloxy) acetamido) benzoic acid (NBA) differentially affect the NMDA concentration response relationship. The inhibition of NMDAR-mediated Ca^2+^ flux by BPN was fully reversible by a process composed of a fast and slow phase. The rapid recovery from BPN was mediated by NMDAR trafficking. Finally, we show that BPN inhibits TRPM4-dependent spontaneous network activity. These results describe novel pharmacological actions of BPN on NMDAR function.

## Materials and Methods

### Reagents

Materials were obtained from the following sources: Fura-2 AM (F1201), DMEM (31053), Hanks’ balanced salt solution (14175), fetal bovine serum (26140), horse serum (16050), penicillin/streptomycin (15140), and poly-D-lysine (A3890401) were from Thermo Fisher Scientific (Carlsbad, CA, USA); Tetanus Toxin, *Clostridium tetani* (582243), NMDA (M3262), glycine (G6388), MK-801 (M107), CNQX (C239), laminin (SIAL-L2020), HEPES (H4034), and DMSO (276855) were from Millipore Sigma (St. Louis, MO, USA); pluronic f-127 (20050) was from AAT Bioquest (Pleasanton, CA, USA); bicuculline (10910), picrotoxin (11281), and Go 6983 (2285) were from Tocris-Biotechne (Minneapolis, MN, USA); Tetrodotoxin (TTX) (14964), bafilomycin A1 (11038), and roscovitine (10009569) were from Cayman Chemical (Ann Arbor, MI, USA); NBA and BPN were from MedChemExpress (Monmouth Juncton, NJ, USA).

### Cell Culture

All animal care and experimental procedures were performed following the Guide for the Care and Use of Laboratory Animals published by the U.S. National Institutes of Health. Ethical approval was granted by the Institutional Animal Care and Use Committee of the University of Minnesota (protocol 1612–34372 A). Rat hippocampal cultures were prepared as previously described (Casby et al. 2024). Briefly, pregnant Sprague-Dawley rats (RGD_737891; Charles River, Wilmington, MA, USA) were euthanized by CO_2_ inhalation, embryonic day 17 fetuses of both sexes were decapitated with sharp scissors, and hippocampi dissected. Tissue was dissociated by trituration. Glass coverslips (number 1.5, 25 mm diameter, catalog number GG-25-15, Neuvitro Corporation, Camas, WA, USA) or glass bottom 24-well plates (catalog number P24G-1.5–10.5-F MatTek, Ashland, MA, USA) were prepared in advance by coating with phosphate buffered saline (PBS) containing 9 µg/mL high molecular weight poly-D-lysine and 0.4 µg/mL laminin, and then cell suspension was plated as a 200 µL droplet at a density of 150,000 cells per coverslip or a 200 µL droplet at a density of 75,000 cells per well. Cultures were grown in DMEM containing 10% fetal calf serum at 37 °C in 10% CO_2_ for 24 h and then maintained in 10% horse serum without mitotic inhibitors for 14–16 days in vitro. The resulting mixed glial-neuronal culture was composed of 18 ± 2% neurons, 70 ± 3% astrocytes, and 9 ± 3% microglia as indicated by immunocytochemistry (Kim et al. 2011). Mixed cultures provide a more physiological depiction of cell signaling in the brain versus a culture of predominantly neurons. All experiments were performed with cultures from at least 3 dissections with 2–3 technical replicates. The cultures consist of tissue from 8 to 12 animals allowing us to consider each replicate independent.

### [Ca^2+^]_i_ Imaging

 Fura-2-based [Ca^2+^]_i_ imaging was performed as previously described (Casby et al. 2024). Briefly, a coverslip with adhered cells was incubated in HEPES-buffered Hanks’ salt solution (HHSS) containing 5 µM Fura-2 AM in 0.1% pluronic acid for 30–60 min at 37 °C. The coverslip was then washed in HHSS for 15–30 min and placed in a recording chamber (Thayer et al. 1988). HHSS contained the following (in mM): HEPES 20, NaCl 137, CaCl_2_ 1.3, MgSO_4_ 0.4, MgCl_2_ 0.5, KCl 5.0, KH_2_PO_4_ 0.4, Na_2_HPO_4_ 0.6, NaHCO_3_ 3.0, and glucose 5.6, pH 7.45. To mimic the aberrant NMDAR-signaling seen in neurodegenerative diseases a Mg^2+^-free solution was used during recordings. This approach relieves the Mg^2+^ blockade of the NMDAR as used previously (Robinson et al. 1993; Baron et al. 2003; Wang et al. 2003).

The recording chamber was placed on the stage of an Olympus IX71 inverted microscope and cells viewed with an Olympus 40x oil immersion objective (UApo 340, 1.35 numerical aperture). Excitation wavelength was selected with a galvanometer-driven monochromator (8-nm slit width) coupled to a 75-W xenon arc lamp (Optoscan; Cairn Research). Fluorescence images (510/40 nm) were projected onto a cooled charge-coupled device camera (Cascade 512B; Roper Scientific) controlled by MetaFluor software (Molecular Devices).

[Ca^2+^]_i_ was monitored using sequential excitation of Fura-2 at 340 and 380 nm; image pairs were collected every 1 s. Neurons were identified based on morphology, for example, size of cell body (soma) presence of dendrites, pyramidal shape, etc. as well as their physical placement above the layer of astrocytes. Cells were superfused at a rate of 1–2 mL per min from gravity-fed reservoirs containing HHSS with the indicated drugs. Applied drugs must cross a large area over the recording chamber making these experiments unideal for interpretation of drug kinetics. A field of view may have between 2 and 15 neurons present (Fig. S1). Calculations were performed by averaging all cells in the field. Traces in figures are a chosen representative trace.

### Immunocytochemistry

Immunocytochemistry (ICC) was performed using a modified version of the receptor internalization assay described by Wang et al. (Wang et al. 2018). Cultured neurons were incubated with a monoclonal GluN2B antibody (1:250, ABCAM catalog # ab93610) for 10 min in PBS. The primary antibody was removed and replaced with fresh culture media containing the drugs of interest, and returned to the incubator at 37 °C for 30 min. Cells were fixed using 4% paraformaldehyde for 10 min, washed with PBS, and then treated for 30 min in blocking buffer (PBS with 2.5% bovine serum albumin). Alexa 488-conjugated goat anti-mouse antibody (1:250) was applied in blocking buffer for 10 min to label surface GluN2B-containing NMDARs. Cells were washed 3 times in PBS and then permeabilized in blocking buffer containing 0.1% tween20 for 30 min. Tween20-containing blocking buffer was removed and cells were quickly rinsed with blocking buffer before adding Cy3 AffinPure fragment donkey anti-mouse antibody (1:250) in blocking buffer for 1 h. Cells were washed in PBS 3 times and stored in PBS at 4 °C for imaging. PBS contained the following (in mM): NaCl 137, KCl 2.7, Na_2_HPO_4_ 10, and KH_2_PO_4_ 1.8 [pH 7.4]. Cells were imaged using a Nikon A1 laser scanning confocal microscope with a 60 × (1.4 numerical aperture) oil-immersion objective as previously described with some modifications (Zhang and Thayer 2018). Briefly, 1 μm steps in the z-dimension were collected spanning 4 μm (5 steps). The image plane with the clearest defined puncta on the green channel was selected for analysis. The same imaging parameters were used for an entire 24 well plate and 3 image fields were taken per well and 3 wells were used per treatment.

### Experimental Design and Statistical Analysis

[Ca^2+^]_i_ was calculated using MetaFluor software (Molecular Devices). The neuronal cell body was selected as the region of interest for all recordings. All neurons within the imaging field were included in the analysis and no exclusions were made. For time course experiments, coverslips from the same cell culture plating were treated in parallel and each coverslip imaged only once. [Ca^2+^]_i_ values are reported as background corrected 340 nm/380 nm fluorescence intensity ratios (F_340_/F_380_).

For ICC experiments, the ratio of surface to internal GluN2B-containing NMDARs was calculated using Image J (Fiji, imagej.net) (Schindelin et al. 2012). For each channel, the average intensity of the entire image was subtracted as background. The neuronal cell body was outlined by referencing the brightfield image. The integrated intensity of the green channel was divided by the integrated intensity of the red channel for every neuronal soma in the field.

Statistical analyses were performed using Prism 10 (GraphPad Software). Primary neuronal cultures were derived from 8 to 12 animals with a unique network forming on each coverslip; thus, each recording from a single coverslip constitutes a biological replicate. For ICC, each soma was considered an N of 1. All data were tested for deviation from a normal distribution with the D’Agostino-Pearson test. For experiments with two groups, a Student’s t test was performed. For experiments with 3 or more groups, a one-way or two-way ANOVA was performed with Tukey’s post-hoc test. Statistical significance was defined as *p* < 0.05. All error bars are shown as SD unless otherwise specified.

## Results

### BPN and NBA Affect the NMDA Concentration Response Relationship

**Fig. 1:**
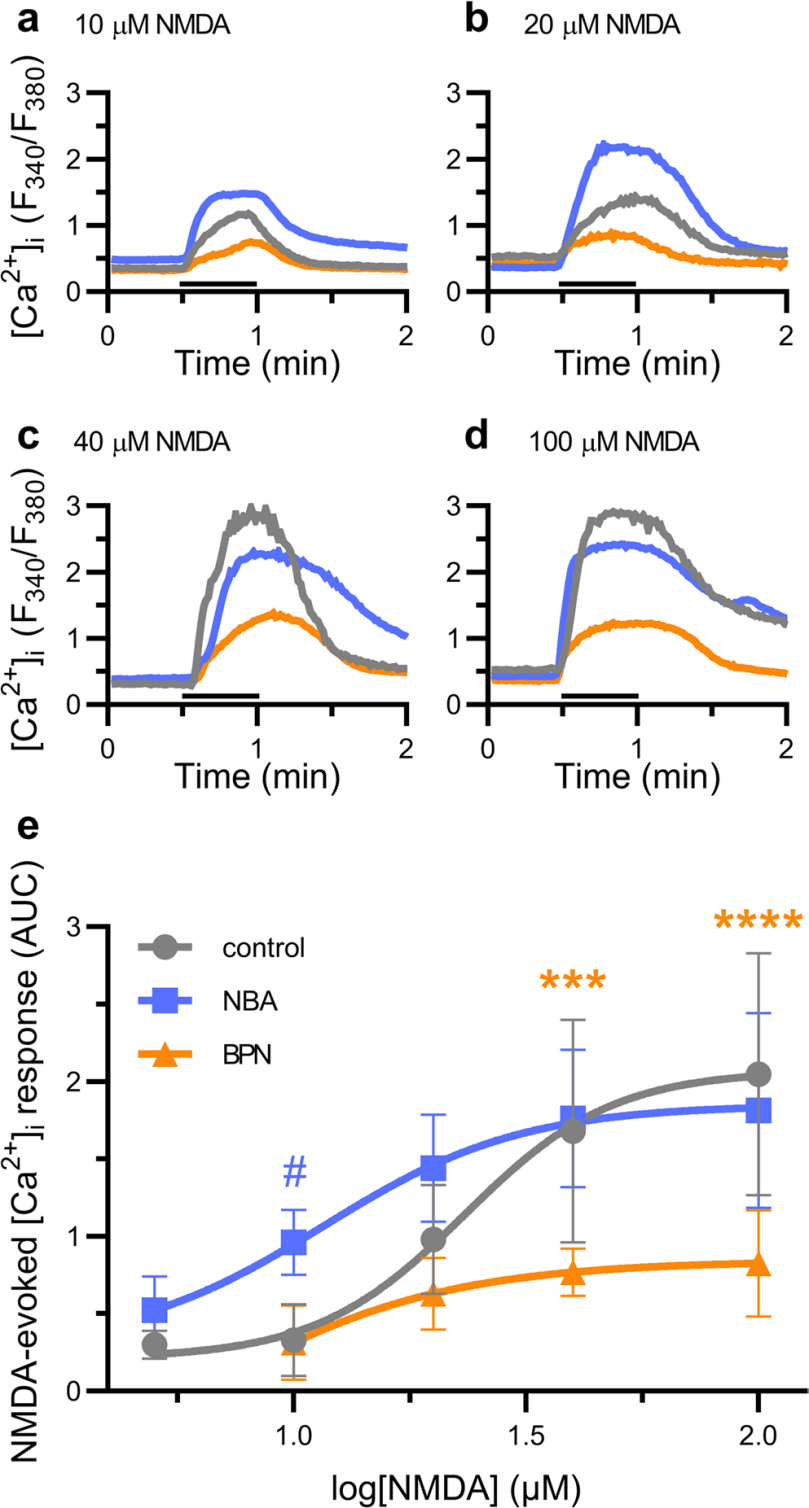
The effects of BPN and NBA on the concentration-dependence of NMDA-evoked increases in [Ca^2+^]_i_ . (**a**-**d**) NMDA-evoked [Ca^2+^]_i_ responses were recorded at 22 °C from cells pre-incubated in Mg^2+^-free HHSS containing 100 nM TTX for 15 min in the absence (control; grey line) or presence of 10 µM BPN (orange line) or 10 µM NBA (blue line). NMDA and glycine (200 µM) were applied in Mg^2+^-free HHSS at the time indicated by the horizontal bar. (**a**) 10 µM NMDA. (**b**) 20 µM NMDA. (**c**) 40 µM NMDA. (**d**) 100 µM NMDA. (**e**) Concentration response summarizes magnitude of the NMDA-evoked [Ca^2+^]_i_ responses (AUC) for replicates of the experiments shown in a-d. Two-way ANOVA: Effect of Drug F_(2,82)_ = 25.90, *p* < 0.0001, effect of NMDA concentration F_(3,82)_ = 22.95, *p* < 0.0001, Drug x NMDA concentration interaction F_(6,82)_ = 3.437, *p* = 0.0045. #*p* < 0.05, ***p < 0.001, ****p < 0.0001 compared to control with Tukey’s post-hoc test. The responses evoked by 5 µM NMDA were used for curve fitting control and NBA datasets but not tested in the presence of BPN and thus excluded from the two-way ANOVA. Concentration-response curves were fit with a sigmoidal equation of the form: change in AUC = A_1_ + [(A_2_-A_1_)/(1 + 10^((logx_0_-x)_*_p)], where x_0_ = EC_50_, x = log[drug], A_1_ = lower bound, A_2_ = upper bound, and p = Hill Slope. Control: A_1_ = 0.24 ± 0.18, A_2_ = 2.1 ± 0.2, x = 23 ± 3.5, *p* = 3.4 ± 1.7. R^2^ goodness of fit: 0.67. NBA: A_1_ = 0.29 ± 0.57, A_2_ = 1.9 ± 0.2, x = 11.6 ± 5.2, *p* = 2.0 ± 1.5. R^2^ goodness of fit: 0.67. In the presence of BPN the data were not well described by a sigmoidal curve fit. R^2^ goodness of fit: 0.43

In a previous study we found that at 22 °C 10 µM BPN inhibited and 10 µM NBA potentiated Ca^2+^ influx evoked by application of 20 µM NMDA and 200 µM glycine to hippocampal neurons in culture (Casby et al. 2024). To investigate how these drugs affect the sensitivity to NMDA, we measured [Ca^2+^]_i_ using fura-2-based digital imaging during 30 s application of 10, 20, 40, or 100 µM NMDA in the presence of 200 µM glycine (Fig. 1a-d). Cells were incubated with 5 µM Fura-2 AM for 30–60 min and washed for 15 min in Mg^2+^ -free HHSS with 100 nM TTX containing no additions (control, grey line), or in the presence of 10 µM NBA (blue line) or 10 µM BPN (orange line). The control NMDA concentration-response relationship was well fit by a sigmoidal curve with an EC_50_ of 23 ± 3 µM, in good agreement with prior reports (Fig. 1e) (Blevins et al. 1995; Hu and Ticku 1995). NBA shifted the concentration-response curve to the left, but did not increase the maximal NMDA-evoked [Ca^2+^]_i_ response at high NMDA concentrations. NBA did not change the maximum NMDA response, but did significantly affect the potency of NMDA, shifting the EC_50_ to 12 ± 5 µM (Student’s t test t_(78)_ = 2.07, *p* = 0.042). In contrast, BPN markedly reduced the maximal NMDA-evoked response. This reduction in the efficacy of NMDA is consistent with non-competitive inhibition.

### BPN Inhibition of NMDA-evoked [Ca^2+^]_i_ Increases is Reversible

**Fig. 2:**
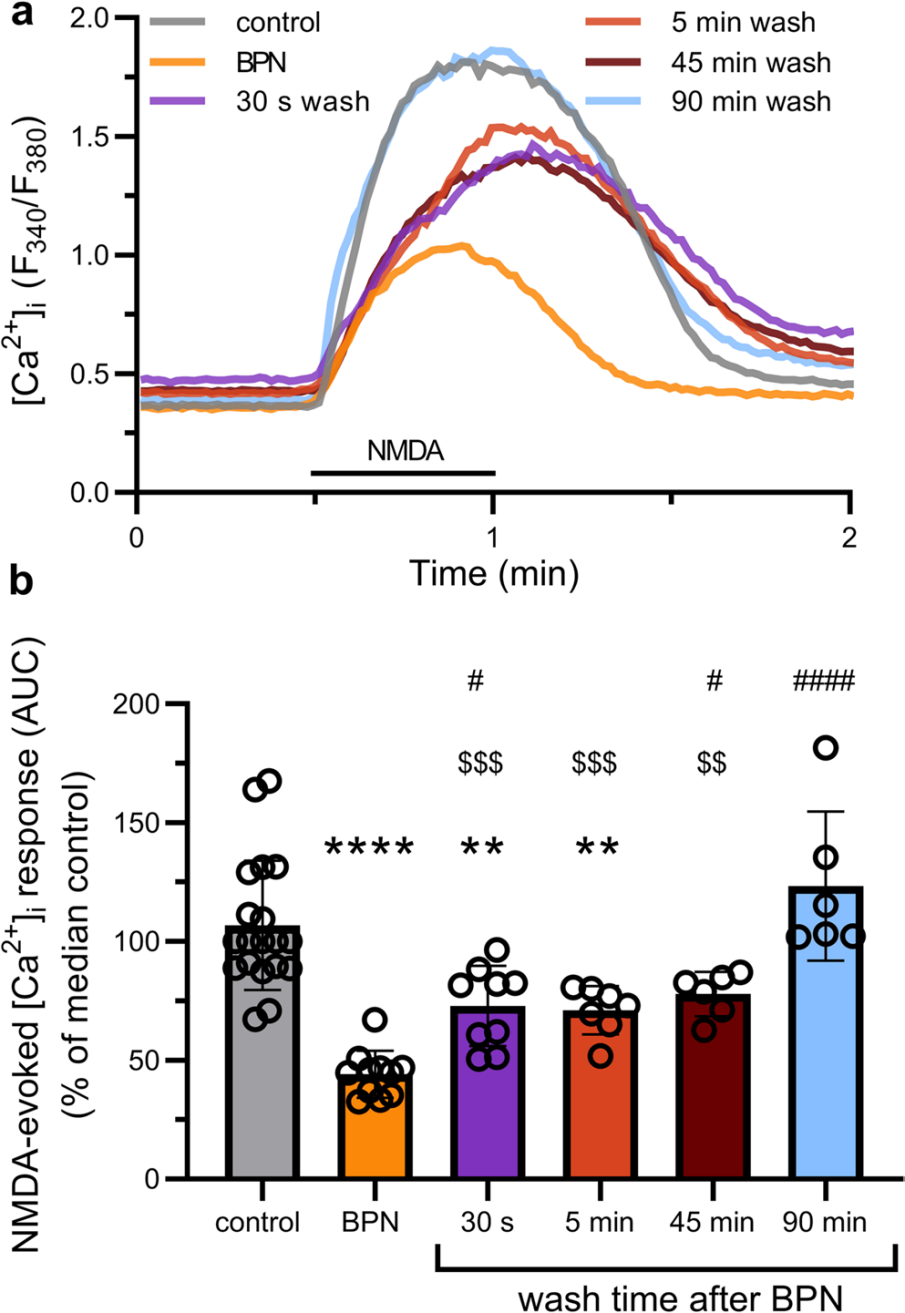
BPN inhibition of NMDA-evoked [Ca^2+^]_i_ responses is rapidly reversible (**a**) NMDA-evoked [Ca^2+^]_i_ responses were recorded at 22 °C from cells pre-incubated in Mg^2+^-free HHSS containing 100 nM TTX for 15 min in the absence (control; grey line), or presence of 10 µM BPN (orange line), or following washout of BPN for 30 s (purple line), 5 min (red line), 45 min (dark red line), or 90 min (blue line). NMDA (20 µM) and glycine (200 µM) were applied in Mg^2+^-free HHSS at the time indicated by the horizontal bar. (**b**) Bar graph summarizes mean NMDA-evoked [Ca^2+^]_i_ responses (AUC) from neurons following no treatment (control; grey bar), 10 µM BPN (orange bar), or following a washout from BPN treatment for 30 s (purple bar), 5 min (red bar) 45 min (dark red bar), or 90 min (blue bar). AUC responses were normalized to the median control response for recordings performed on the same day. One-way ANOVA F_(5,52)_ = 18.00, *p* < 0.0001. **p < 0.01, ****p < 0.0001 compared to control; #*p* < 0.05, ####*p* < 0.0001 compared to BPN; $$*p* < 0.01, $$$*p* < 0.001 compared to 90 min wash with Tukey’s post hoc test

Because BPN acted as a noncompetitive inhibitor of NMDARs, and some noncompetitive NMDAR inhibitors, such as pore blockers, are slowly reversible (Wilcox et al. 2022), we next wanted to know if the effect of BPN was readily reversible. To test this idea (Fig. 2), cells were incubated with fura-2 AM for 30–60 min and washed for 15 min in Mg^2+^ -free HHSS with 100nM TTX containing no additions (control, grey line) or addition of 10 µM BPN (orange line). Cells treated with 10 µM BPN were washed with Mg^2+^ -free HHSS with 100nM TTX for 30 s (purple line), 5 min (red line), 45 min (dark red line) or 90 min (blue line) before application of 20 µM NMDA and 200 µM glycine for 30 s. BPN treated cells exhibited a [Ca^2+^]_i_ response that was 41 ± 7% of the control response. After only a 30 s wash in the absence of BPN, cells recovered to 68 ± 8% of the control response, which was similar to that seen after washing for 5 min (67 ± 9%) or 45 min (73 ± 9%). 90 min following BPN removal the response increased further to 116 ± 9% compared to control (Fig. 2). These results suggest that upon washout of BPN there is a rapid initial recovery of the NMDA-evoked increase in [Ca^2+^]_i_ followed by a slower return to full response magnitude.

### Reversal of BPN Inhibition of NMDA-Evoked Increases in [Ca^2+^]_i_ is Dependent on Receptor Trafficking

**Fig. 3:**
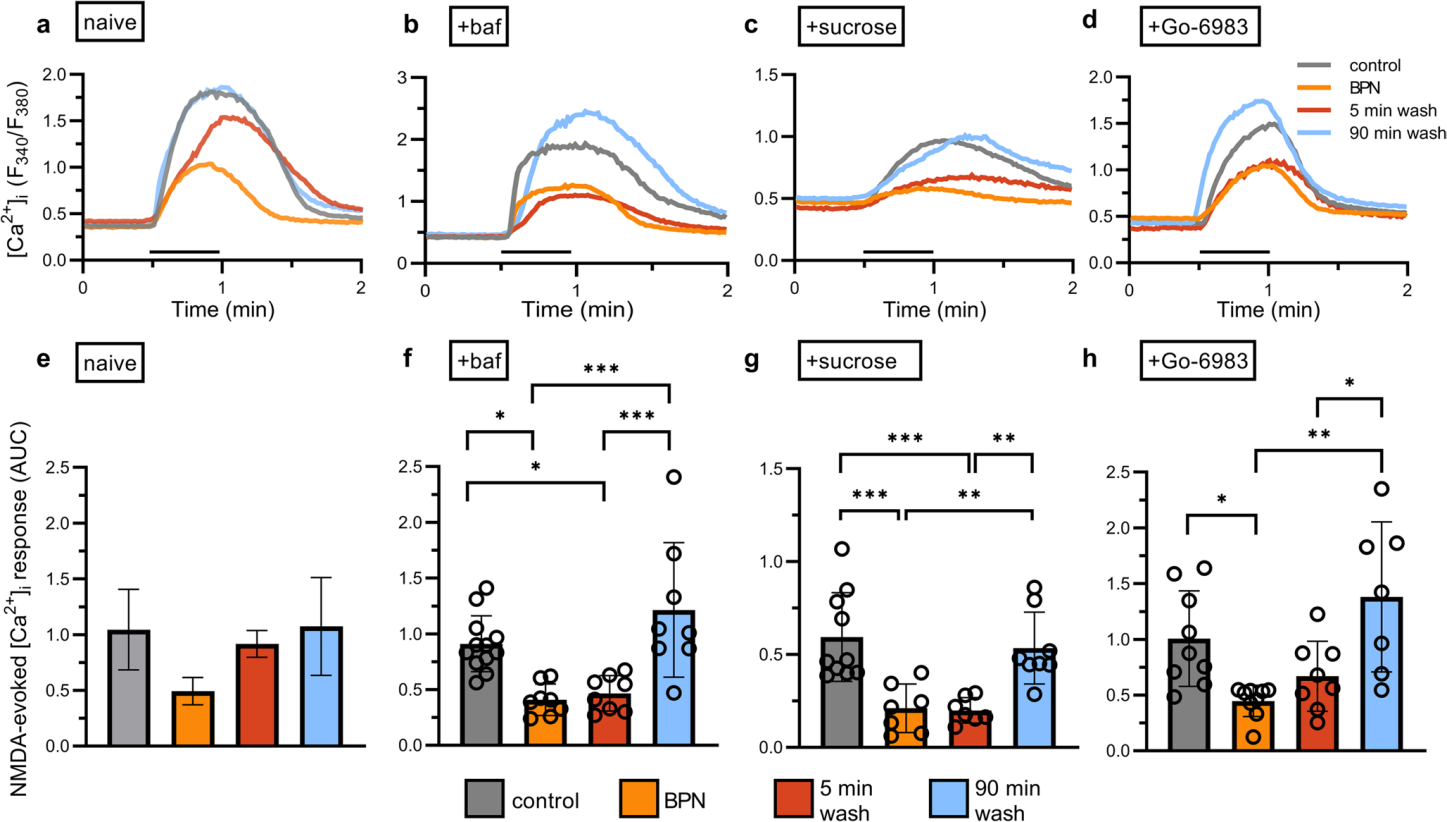
Drugs that inhibit receptor trafficking slow recovery from BPN treatment (**a**-**d**) NMDA-evoked [Ca^2+^]_i_ responses were recorded at 22 °C from cells pre-incubated in Mg^2+^-free HHSS containing 100nM TTX for 15 min in the absence (control; grey line), presence of 10 µM BPN (orange line), or following a washout of BPN treatment for 5 min (red line) or 90 min (blue line). NMDA and glycine (200 µM) were applied in Mg^2+^-free HHSS at the time indicated by the horizontal bar. Graphs of experimentally naïve cells from Fig. 2 were added for easy comparison (**a** and **e**). Cells were pretreated before BPN exposure with (**b**) 100 nM baf for 45 min, (**c**) 450 mM sucrose for 45 min, or (**d**) 500 nM Go-6983 for 30 min. (**e**-**h**) Bar graphs summarize mean NMDA-evoked [Ca^2+^]_i_ responses (AUC) from neurons in the absence (control; grey bar) or presence of 10 µM BPN (orange bar), or following a washout from BPN treatment for 5 min (red bar) or 90 min (blue bar). Cells were pretreated before BPN exposure with (**f**) 100 nM baf for 45 min, (**g**) 450 mM sucrose for 45 min, or (**h**) 500 nM Go-6983 for 30 min. Treatments were maintained throughout the recording. Baf treatment one-way ANOVA F_(3,32)_ = 10.79, *p* < 0.0001. Sucrose treatment one-way ANOVA F_(3,28)_ = 10.86, *p* < 0.0001. Go-6983 treatment one-way ANOVA F_(3,29)_ = 7.493, *p* = 0.0007. *p < 0.05, **p < 0.01, ***p < 0.001 with Tukey’s post hoc test

There is precedent for NMDAR binding partners to facilitate receptor trafficking (Zhou et al. 2021) (Zong et al. 2025), and thus we hypothesized that recovery from BPN inhibition might involve NMDARs unbound from TRPM4 being endocytosed during BPN application, followed by trafficking of new NMDAR/TRPM4 complexes to the surface after BPN washout. To test this hypothesis, we pretreated with 100 nM bafilomycin A1 (baf) 30 min before application of 10 µM BPN, and maintained baf throughout the entire experiment (Fig. 3a and d). Baf inhibits the proton pump vacuolar-type H^+^ ATPase (V-ATPase) that is responsible for acidifying lysosomes, endosomes, and vesicles. Baf blocks the transport from early to late endosomes by preventing the acidic environment needed to recycle receptors to the cell surface (Bayer et al. 1998). As shown in Fig. 3b, responses from baf treated control cells (grey line) were not significantly different from NMDA-evoked [Ca^2+^]_i_ responses recorded from naive control cells (control from Fig. 2b compared to control in Fig. 3d; Student’s t test: t_(32)_ = 1.138, *p* = 0.2637). Application of baf prevented the rapid recovery from BPN treatment, in contrast to that observed in untreated control cells after a 5 min washout. However, cells did make a full recovery by 90 min (Fig. 3a blue line). In the presence of baf, BPN (Fig. 3a orange line) reduced the [Ca^2+^]_i_ response to 45 ± 17% of control and after 5 min washout of BPN (Fig. 3A red line), in the continued presence of baf, the response remained at 51 ± 17% of control. Thus, in the presence of baf washout of BPN failed to produce statistically significant recovery of the NMDA-evoked response at this early time point (Fig. 3d). Washout of BPN for 90 min restored the response to 133 ± 17% of control (Fig. 3d).

The results from baf application indicated the initial rapid recovery is dependent on receptor trafficking through endosomes. This is consistent with the known rapid endosomal recycling of GluN2B-containing NMDARs (Lavezzari et al. 2004). To follow up, we tested treatments that inhibit both endocytosis and exocytosis. 450 mM sucrose has been shown previously to inhibit NMDAR endocytosis (Minnis et al. 2003; Nong et al. 2003). 450 mM sucrose was applied 30 min before BPN exposure and maintained throughout the experiment. In the presence of high sucrose BPN (Fig. 3b, orange line) inhibited the [Ca^2+^]_i_ response to 36 ± 15% of control and the 5 min washout (Fig. 3b, red line) did not restore the response to control levels (33.5 ± 15% of control). However, 90 min washout of BPN resulted in recovery to 90 ± 14% of control (Fig. 3b, blue line). Thus, treatment with high sucrose prevented the rapid recovery from BPN treatment observed in untreated control cells (Fig. 3e). The 450 mM sucrose treated control cells (Fig. 3b, grey line) showed a decrease in [Ca^2+^]_i_ response relative to that observed in untreated control cells (control from Fig. 2b compared to control in Fig. 3e; Student’s t test: t_(30)_ = 3.583, *p* = 0.0012). The inhibition of rapid recovery by high sucrose is consistent with a role for recycling endosomes in the recovery from BPN. However, the reduced amplitude of the control response in high sucrose complicates interpretation of treatments that alter endocytosis.

Protein kinase C (PKC) increases NMDAR trafficking to the cell surface (Lan et al. 2001). In Fig. 3c we show the effects of inhibiting PKC-dependent NMDAR exocytosis using the PKC inhibitor Go-6850 (500 nM). Go-6850 was applied 30 min before BPN exposure and maintained throughout the experiment. Go-6850 did not affect the magnitude of the control response (control from Fig. 2b compared to control in Fig. 3f; Student’s t test: t_(29)_ = 0.2550, *p* = 0.8005). BPN remained effective in the presence of Go-6850; BPN reduced the [Ca^2+^]_i_ response to 38 ± 21% of control. In the presence of Go-6850 the rapid recovery from BPN treatment was attenuated, recovering to 54 ± 22% of control. The response after 5 min washout of BPN in the maintained presence of Go-6850 was not statistically different from either the control or the BPN-treated response. Interestingly, 90 min after washout of BPN the NMDA-evoked increase in [Ca^2+^]_i_ recovered to 123 ± 21% of control, which was significantly larger than the 5 min recovery response (Fig. 3c).

**Fig. 4:**
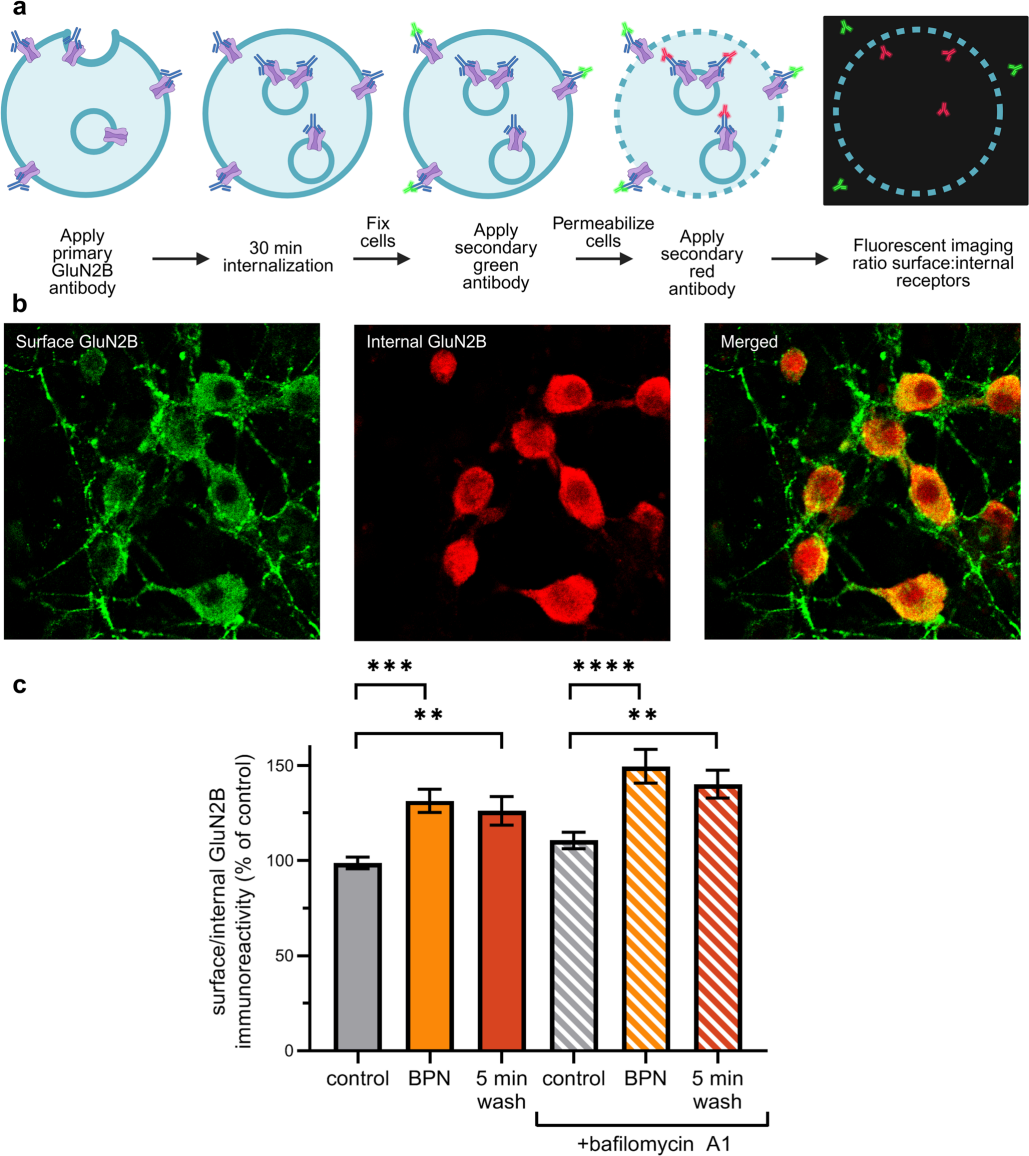
BPN increases surface expression of GluN2B. ICC was used to measure the cell surface and internal expression of the GluN2B subunit of NMDARs as described in methods. (**a**) Schematic of ICC experiment shows labeling of live cells for 10 min with primary monoclonal mouse anti-GluN2B antibody followed by incubation for 30 min to allow internalization. Cells were then fixed and surface primary antibody labeled with green fluorescent secondary antibody followed by permeabilization and labeling with red fluorescent secondary antibody. Fluorescent images were acquired using a confocal microscope as described in Methods. Cell somas were selected from a differential interference contrast image. The integrated intensity for this region was determined for the cell surface (green) and internalized (red) GluN2B immunoreactivity. (**b**) Example images showing surface GluN2B immunoreactivity (green channel), internal GluN2B immunoreactivity (red channel), and a composite of both channels merged. (**c**) GluN2B immunoreactivity was quantified on the cell surface and for internalized GluN2B as described above (**a**-**b**). Surface/Internal ratios for GluN2B immunoreactivity were calculated for cells treated in the absence (solid bars) or presence of 100 nM bafilomycin A1 (striped bars). Cells were incubated for 30 min during the internalization step in culture media in the absence (control; grey bars), presence of 10 µM BPN (orange bars), or following a washout from BPN treatment for 5 min (red bars). Two-way ANOVA: Effect of BPN F_(2,1026)_ = 20.70, *p* = 0.005, effect of baf A1 F_(1,1026)_ = 8.450, *p* = 0.004, BPN x baf A1 interaction F_(2,1026)_ = 0.1407, *p* = 0.87. **p < 0.01, ***p < 0.001, ****p < 0.0001 compared to control with Tukey’s post-hoc test. Error bars = SEM

Because NMDAR trafficking through endosomes appears to be responsible for the rapid phase of recovery from BPN we used an ICC-based membrane internalization assay (Wang et al. 2018) to follow NMDARs during BPN treatment and recovery (Fig. 4a). This assay labeled surface GluN2B immunoreactivity with a green fluorescent secondary antibody and internalized GluN2B immunoreactivity with a red fluorescent secondary antibody (Fig. 4b). For each soma in the field of view the ratio of the surface to internalized label (green/red) was calculated. BPN (10 µM) increased the level of GluN2B immunoreactivity on the cell surface relative to internal GluN2B. Because NMDAR function was markedly inhibited after this treatment, we conclude that the increased NMDARs on the cell surface are not fully active because of the presence of BPN. The increased surface expression of GluN2B immunoreactivity could be a homeostatic process similar to the increased forward trafficking of NMDARs observed during activity suppression (Skeberdis et al. 2001; Lin et al. 2006). To validate this effect was not due to a change in overall expression of GluN2B, The total immunoreactivity of GluN2B was summed and we found BPN treated cells had an overall reduction in expression of GluN2B by 11.9 ± 5% (Fig. S3). While this value represents a significant decrease in expression, we do not believe this modest decrease is a main contributor to the difference in the ratio of surface to internal expression we report. Following washout of BPN the surface/internal ratio of GluN2B immunoreactivity stayed elevated. In the presence of baf the initial control distribution of receptors was unchanged relative to untreated control. BPN induced a significant increase in surface relative to internal GluN2B immunoreactivity. The most parsimonious explanation of these results is that BPN does not directly interfere with NMDAR trafficking but rather the drug’s inhibition of NMDAR function drives compensatory changes. Furthermore, the results shown in Figs. 3 and 4, when taken together, indicate that trafficking mediated insertion of functional NMDAR complexes into the surface membrane mediates the rapid recovery phase following BPN washout.

### BPN Affects TRPM4-Dependent Spontaneous Network Activity

**Fig. 5:**
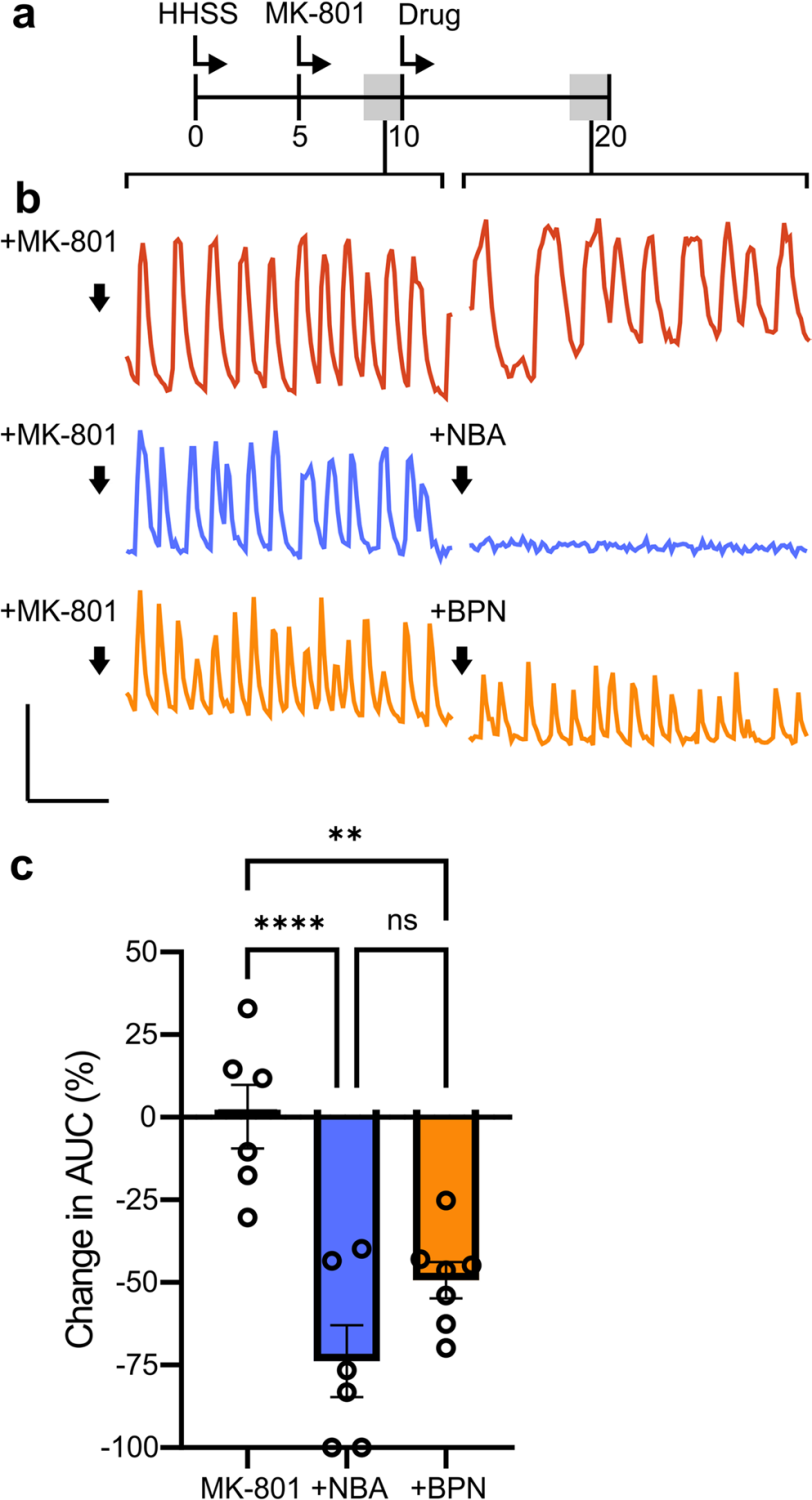
BPN partially inhibits TRPM4-dependent spontaneous activity (**a**) Timeline of pretreatment and imaging for spontaneous activity experiments. Shading denotes epochs that were quantified and selected for representative traces. (**b**) Representative traces for fura-2-based digital imaging of the spontaneous activity of neurons treated with 10 µM MK-801 (red line), 10 µM MK-801 and 10 µM NBA (blue line), or 10 µM MK-801 and 10 µM BPN (orange line). (**c**) Bar graph summarizing the change in spontaneous activity (AUC) of cells treated with 10 µM MK-801 after no additions (control, grey bar), 10 µM NBA (blue bar), or 10 µM BPN (orange bar). One-way ANOVA F_(2,16)_ = 18.47, *p* < 0.0001. **p < 0.01, ****p < 0.0001 with Tukey’s post hoc test. Scale bars = 0.1 F_340_/F_380_ by 30 s

We next determined if BPN affected TRPM4 function. Primary hippocampal cultures exhibit spontaneous synaptic activity that produces recurring [Ca^2+^]_i_ spikes. Because TRPM4 has been shown to increase excitability we used [Ca^2+^]_i_ spiking as an indirect assay for TRPM4 function. We used the selective TRPM4 inhibitor NBA (10 µM) (Delalande et al. 2019) to define the role of TRPM4 in maintaining [Ca^2+^]_i_ spiking activity. Cells were incubated with 5 µM fura-2 AM and washed for 15 min in HHSS containing physiological Mg^2+^ (0.9 mM). NBA (10 µM) treatment reduced Ca^2+^ spiking but also elevated the baseline suggesting an incomplete inhibition of activity coupled with a potentiation of Ca^2+^ influx via NMDARs (Fig. S2) in agreement with the results shown in Fig. 1 and our previous finding that NBA potentiates NMDARs at room temperature (Casby et al. 2024). Thus, to evaluate the effects of drugs acting on TRPM4 in the absence of NMDARs we first treated the culture with the NMDAR inhibitor MK-801 (10 µM). In the combined presence of MK-801 and 10 µM NBA we observed a loss (73.8 ± 11%) of activity (Fig. 5). From this we can conclude that the Ca^2+^ signaling taking place in the presence of MK-801 is TRPM4-dependent. We then tested 10 µM BPN in the presence of MK-801 to determine if BPN affects TRPM4-dependent spontaneous network activity. Over multiple recordings, we observed BPN reducing the amplitude of the peaks, the number of peaks, or both (Fig. 5). To quantify our observations, we measured the area under the curve (AUC), to capture both changes in frequency and changes in amplitude, for 2 min of recording immediately prior to NBA or BPN application and compared it to the AUC for the last 2 min of recording in the 10 min experiment (after 8 min of drug application). We found that while BPN did not completely block the TRPM4-dependent spontaneous network activity, it did significantly inhibit it by 49 ± 6%.

## Discussion

Here, we characterized the effects of the NMDAR/TRPM4 interface inhibitor BPN and the potent and selective TRPM4 inhibitor NBA on NMDAR and TRPM4 function. BPN acted non-competitively to inhibit NMDA-evoked increases in the [Ca^2+^]_i_ and NBA increased the potency of NMDA but not maximum effect size, suggesting that TRPM4 modulates the NMDAR allosterically. We next explored the reversibility of BPN inhibition of NMDAR function, finding that NMDAR activity was rapidly restored upon washout of BPN via recycling of GluN2B-containing NMDARs to the cell surface. Finally, we examined the effects of BPN on TRPM4 function. Because TRPM4 modulates neuronal network activity by enhancing depolarization (Menigoz et al. 2016), we used network driven Ca^2+^ spiking activity as an assay and showed for the first time that an NMDAR/TRPM4 interface inhibitor also inhibited TRPM4 function.

Both BPN and NBA modulate NMDAR-mediated Ca^2+^ influx (Casby et al. 2024). The nature of this modulation provides insight into the TRPM4/NMDAR interaction. BPN inhibited NMDA evoked responses in a non-competitive manner (Fig. 1), consistent with biochemical studies that showed that BPN disrupts the NMDAR/TRPM4 complex (Yan et al. 2020). The inhibition of the NMDA-evoked Ca^2+^ response by 10 µM BPN, which is a supramaximal concentration (Yan et al. 2020), was not complete. Several mechanisms might account for this residual activity. NMDARs might retain some function in the absence of bound TRPM4. Alternatively, the remaining activity might reflect NMDARs bound to other partners such as TRPM2 or α2δ−1 (Chen et al. 2018; Zong et al. 2025). It is not clear how the various NMDAR binding partners interact or whether they co-localize to the same regions of the cell. For example, TRPM4 is thought to preferentially bind esNMDARs (Yan et al. 2020), while α2δ−1 is known to bind sNMDARs (Chen et al. 2018). GluN2B-containing NMDARs rapidly cycle through endosomes (Lavezzari et al. 2004); perhaps steady-state function results from the rate of new NMDAR/TRPM4 inserted into the membrane balanced by the rate of BPN inhibition of function. The observation that binding to TRPM4 is required for maximal NMDAR function indicates that TRPM4 modulates the NMDAR allosterically. Allosteric modulation of NMDAR by TRPM4 is further supported by our finding that the TRPM4 inhibitor NBA increased the potency of NMDA. Since NBA binds to TRPM4, and our previous study showed that ion flux through TRPM4 was not required for TRPM4 modulation of NMDAR function, the binding of NBA must cause a structural change in TRPM4 that then causes an allosteric change in the NMDAR. NBA potentiates NMDARs at room but not physiological temperature (Casby et al. 2024), in good agreement with temperature-dependent changes in TRPM4 structure and pharmacology (Hu et al. 2024). The pharmacological effects of drugs acting on TRPM4 highlight the complexity of the interaction between TRPM4 and NMDARs.

Because BPN disrupts the NMDAR/TRPM4 complex and is a non-competitive inhibitor of NMDA-evoked responses, we explored the reversibility of BPN’s effects. After washout of BPN, NMDAR function recovered in two phases, an initial rapid recovery followed by a slower return to control levels. The rapid phase occurred over approximately 30 s and required recycling endosomes. The most compelling evidence supporting our contention that NMDARs were inserted into the membrane to mediate this fast phase of recovery was its effective blockade by baf. Inhibiting endocytosis with high sucrose and inhibiting exocytosis with Go-6850 were consistent with this hypothesis. However, the effects of baf were more compelling because it did not affect the control response, it is a selective inhibitor of the H^+^ pump that is needed for maturation of endosomes, and it produced a robust and complete block of the rapid phase of recovery. Our conclusion that trafficking of receptors to the cell surface mediates the fast phase of recovery is consistent with the rapid recycling of GluN2B-containing NMDARs (Lavezzari et al. 2004). 90 min after the removal of BPN NMDAR function fully recovered in the presence of baf, suggesting that the slow phase of recovery is independent of NMDAR trafficking via endosomes. Ligand binding experiments determining a dissociation constant for BPN have not been reported and drugs that access ion channels via the membrane can reverse slowly (Wilcox et al. 2022). However, 90 min in the absence of drug might be sufficient for BPN to dissociate from TRPM4 allowing reassembly of the NMDAR/TRPM4 complex.

While trafficking mediates the rapid phase of recovery from BPN, we did not find evidence that BPN affected trafficking directly. This finding contrasts with the reduced insertion of NMDARs into the cell surface of sensory neurons in the presence of gabapentenoids that disrupt the NMDAR/α2δ−1 complex (Chen et al. 2018). ICC experiments found a higher ratio of surface to internalized GluN2B immunoreactivity in cells treated with BPN even though NMDAR function was inhibited under identical conditions. The most parsimonious explanation for these observations is that in response to reduced NMDAR function in the presence of BPN a homeostatic compensation increases the trafficking of receptor complexes stored in endosomes that are poised for membrane insertion. There is precedent for increased forward trafficking of NMDARs during activity suppression (Skeberdis et al. 2001; Lin et al. 2006). The inhibition of recovery from BPN inhibition of NMDAR function by baf along with a lack of return to the initial ratio of surface to internalized NMDAR in baf, is consistent with a pool of functional NMDAR/TRPM4 complexes in endosomes that are inserted into the cell surface during BPN treatment that are then inhibited when they reach the cell surface by the presence of excess extracellular drug. A hypothetical scheme that accounts for the effects of BPN on NMDAR function and localization is shown in supplementary figure S4.

Little is known about how the NMDAR affects TRPM4 function. It is possible dissociating TRPM4 from the NMDAR could reduce Ca^2+^-dependent activation of TRPM4 by distancing it from the mouth of the NMDAR ion channel. Furthermore, BPN was discovered through a screen of compounds that bind to TwinF, an intracellular domain of TRPM4 that interacts with an intracellular domain of GluN2A and GluN2B. Thus, it is possible that BPN could affect TRPM4 function by direct inhibition of TRPM4 or indirectly by separating it from NMDARs. Because TRPM4 activation depolarizes membrane potential (Launay et al. 2002) which affects Ca^2+^ signaling, we were able to use Ca^2+^ imaging to indirectly monitor TRPM4 activity. We found the spontaneous network signaling occurring in the presence of the NMDAR inhibitor MK-801 was inhibited by the TRPM4 inhibitor NBA. BPN partially but significantly inhibited this TRPM4-dependent Ca^2+^ flux. This novel finding shows that in addition to inhibiting NMDAR function BPN also inhibits TRPM4. Thus, inhibition of TRPM4 may play a role in BPN’s neuroprotective mechanism; this is supported by research showing that inhibition of TRPM4 provides neuroprotection in models of neuronal injury (Bianchi et al. 2018; Dundar et al. 2020; Ma et al. 2023b). However, TRPM4 also has physiological roles as an amplifier that enhances membrane depolarization (Launay et al. 2002). For example, TRPM4 contributes to the depolarization that relieves the Mg^2+^ block of the NMDAR that is required for long-term potentiation of synaptic transmission (Menigoz et al. 2016). In future studies it will be important to determine if TRPM4 affects synaptic plasticity and network function as an individual ion channel or only when in complex with NMDARs. Furthermore, are the channels inhibited by NBA localized to somatodendritic regions and thus broadly affecting excitability or, contrary to some (Yan et al. 2020) but in agreement with other reports (Menigoz et al. 2016), are they located in synaptic spines to modulate synaptic plasticity? TRPM4 also amplifies Ca^2+^ signals created by mechanosensitive channels such as piezo1 (Yoo et al. 2022). The degree to which inhibition of TRPM4 function underlies the therapeutic and adverse effects of NMDAR/TRPM4 interface inhibitors is not clear but certainly action on TRPM4 should be considered.

We acknowledge there are shortcomings of our study. While BPN is an exciting new drug, the field has already created new analogs such as FP802 that are better tolerated in animals (Yan et al. 2024). We focused on BPN as a tool to study the effects of NMDAR/TRPM4 interface inhibitors on NMDAR and TRPM4 function in neurons because it is the prototype of this class of drugs and subsequent drugs were identified through screens that captured the same mechanism (Yan et al. 2024). It is possible that other compounds identified in the same screen could have additional effects. A drawback to assessing NMDAR function by recording changes in the [Ca^2+^]_i_ is that the measurements are influenced by other sources of Ca^2+^ as well as cytoplasmic Ca^2+^ clearance. The advantages of using a non-invasive optical method that leaves intracellular signaling pathways intact outweigh alternative approaches. The indirect measure of TRPM4 activity used here was chosen because TRPM4 currents in primary hippocampal neurons are small and assessing TRPM4 activity from spontaneous network activity amplifies the effects of TRPM4. Thus, the assay, while quantitative, does not directly relate drug effects to the magnitude of ion flux through the channel. A strength of the approach is that the modulation of network activity is highly relevant to CNS function. Because TRPM4 expression is increased in certain disease states (Bianchi et al. 2018; Dundar et al. 2020), an alternative approach might examine the role for TRPM4 under pathological conditions.

The neuroprotection afforded by inhibitors of the NMDAR/TRPM4 interface provides a novel way to treat neurodegenerative diseases and brain injuries. The non-competitive inhibition and temperature-dependent potentiation of NMDA-evoked responses by BPN and NBA, respectively, highlight the complex allosteric regulation of NMDARs by binding TRPM4 and suggest many future experiments exploring how this interaction is affected by the multiple ligands that bind to each of these ion channels. Trafficking of NMDARs contributes to recovery from BPN inhibition; the restoration of function upon removal of BPN may be a particularly useful approach to studying the potential role of TRPM4 in NMDAR trafficking and cell surface stability. The novel finding that BPN inhibits TRPM4-dependent spontaneous Ca^2+^ activity suggests an additional mechanism that may contribute to the pharmacology of NMDAR/TRPM4 interface inhibitors. The NMDAR/TRPM4 interface inhibitors are an exciting new class of neuroprotective drugs that, as shown here, also provide a useful tool to probe the function of NMDARs and TRPM4 channels.

## Supplementary Information

Below is the link to the electronic supplementary material.

Supplementary Material 1 (PDF 613 KB)

## Data Availability

Data are provided within the manuscript and supplementary information files.

## Abbreviations

[Ca^2+^]_i_: intracellular free Ca^2+^concentration
ALS: amyotrophic lateral sclerosis
AUC: area under the curve
baf: bafilomycin A1
BPN: brophenexin
esNMDARs: extrasynaptic *N*-methyl-D-aspartate receptors
HHSS: HEPES-buffered Hanks’ salt solution
ICC: Immunocytochemistry
NBA: 4-chloro-2-(2-(naphthalene-1-yloxy) acetamido) benzoic acid
NMDARs: *N*-methyl-D-aspartate receptors
PBS: phosphate buffered saline
PKCγ: protein kinase C gamma
sNMDARs: synaptic *N*-methyl-D-aspartate receptors
TRPM2: transient receptor potential melastatin 2
TRPM4: transient receptor potential melastatin 4
TTX: tetrodotoxin
V-ATPase: proton pump vacuolar-type H^+^ ATPase
