# Supplementary material for: Modulation of NMDA Receptor and TRPM4 Activity in Hippocampal Neurons with the NMDA Receptor/TRPM4 Interface Inhibitor Brophenexin
Source: Neurotox Res. 2026 Mar 6;44(2):11. doi: 10.1007/s12640-026-00788-0 (PMC12965916; doi:10.1007/s12640-026-00788-0)
Supplement: Supplementary file 1 — Supplementary Material 1 (PDF 613 KB) [file 12640_2026_788_MOESM1_ESM.pdf]

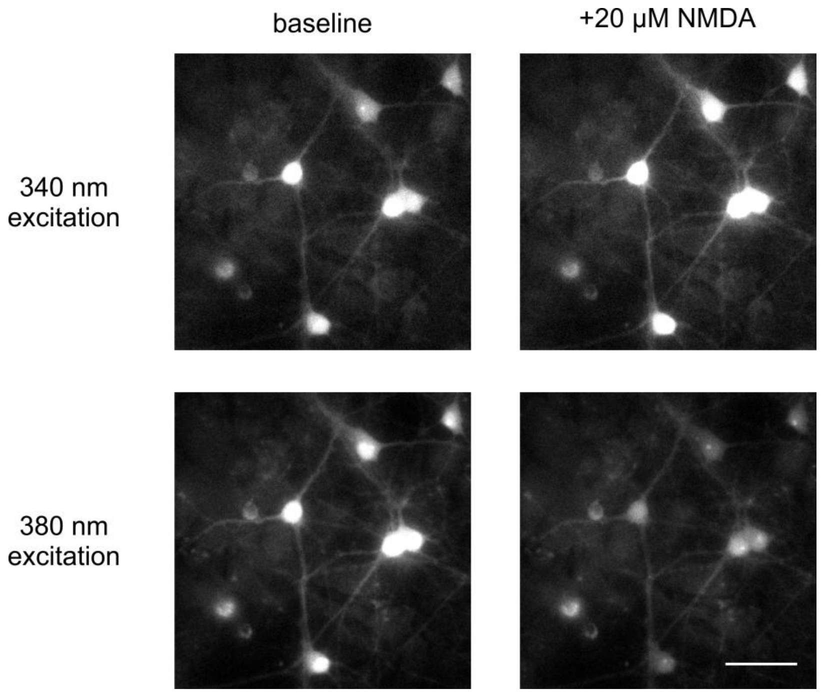

Fig. S1 Representative images of neurons loaded with Fura-2
Cells were excited at 340 nm (Top) and 380 nm (Bottom) once per second over the course of the experiment. Select images were chosen to represent the cells at baseline (Left) and during 20 µM NMDA treatment (Right). Scale bar = 50 µm.

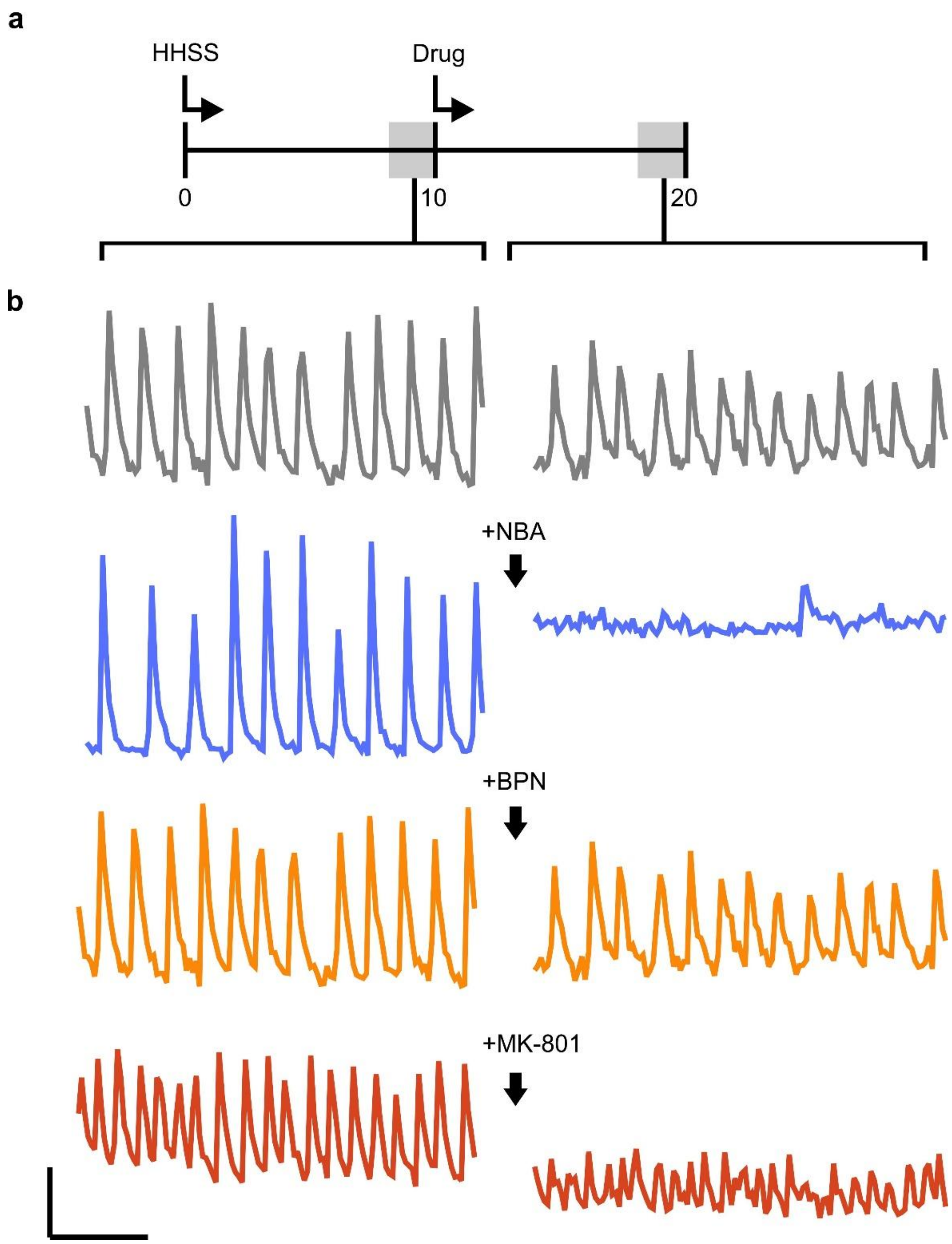

Fig. S2 Spontaneous [Ca2+]i spiking activity has NMDAR- and TRPM4-dependent components

(a) Timeline of pretreatment and imaging for spontaneous activity experiments. Shading denotes epochs selected for representative traces. (b) Representative traces for fura-2-based digital-imaging recordings of the spontaneous activity of neurons before treatment followed by the addition of no treatment (control, grey line), 10 μM NBA (blue line), 10 μM BPN (orange line), or 10 μM MK-801 (red line). Scale bars = 0.1 F340/F380 by 30 s

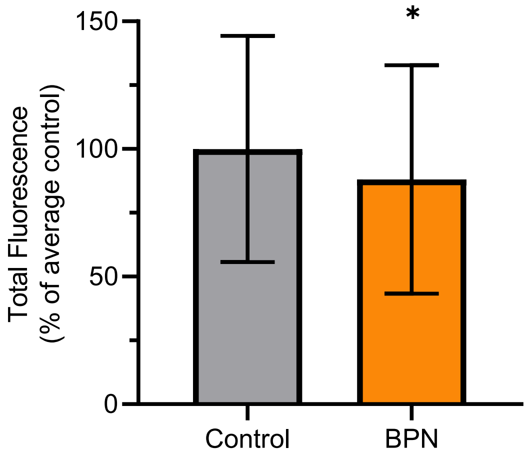

Fig. S3 Total fluorescence of GluN2B immunoreactivity
The total fluorescence of all GluN2B immunoreactivity including both internally and externally expressed receptors was summed for cells in the absence (control; grey bar) or presence of 10 µM BPN (orange bar). The total fluorescence was normalized to the control from the same plating. Student's t-test: $t_{(365)} = 2.560$, $p = 0.0109$. *$p < 0.05$ with Tukey's post hoc test.

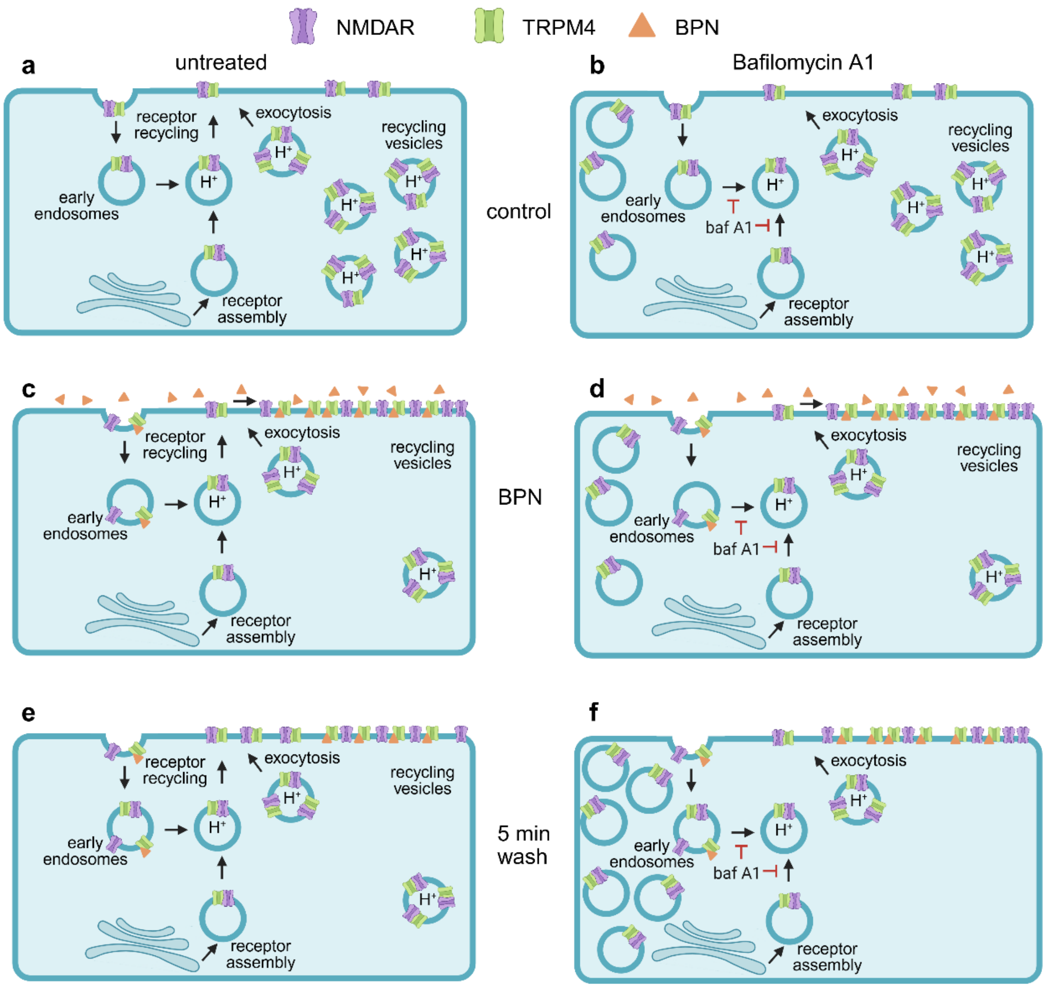

Fig S4 Summary schematic depicts hypothesized trafficking of NMDARs in the absence and presence of BPN

(a) Under control conditions, GluN2B-containing NMDARs recycle rapidly and new receptors are exported from the endoplasmic reticulum. (b) The application of bafilomycin A1 prevents the acidification of endosomes which disrupts recycling. (c) In the presence of BPN receptors newly inserted into the membrane bind BPN and the NMDAR/TRPM4 complex is disrupted. To compensate for the BPN-induced loss of function additional receptors are inserted into the membrane, increasing GluN2B immunoreactivity on the cell surface. (d) In the presence of BPN and baf, recycling vesicles prepared before baf application are available to insert NMDARs into the membrane, consistent with the increase in GluN2B immunoreactivity seen under these conditions. (e) When BPN is removed, we hypothesize it does not dissociate from TRPM4 within 5 min, thus inhibited NMDARs remain at the surface with new functional NMDAR/TRPM4 complexes inserted into the surface membrane, explaining the maintained high GluN2B immunoreactivity and the partial restoration of function. (f) When BPN is removed in the presence of baf, there are no recycling vesicles ready for exocytosis. Instead, early endosomes will start to accumulate waiting for acidification. This explains the maintained high surface GluN2B-immunoreactivity with no recovery of function.
